# Establishment of a New Abdominal Aortic Aneurysm Model in Rats by a Retroperitoneal Approach

**DOI:** 10.3389/fcvm.2022.808732

**Published:** 2022-02-23

**Authors:** Jun-Xing Zhu, Quan-Qiao Tang, Can Zhou, Xing-Chi Shi, Si-Yi Yi, Ying Yang

**Affiliations:** ^1^Department of Clinical Medicine, North Sichuang Medical College, Nanchong, China; ^2^Department of Cardiovascular Disease, Affiliated Hospital, North Sichuang Medical College, Nanchong, China

**Keywords:** abdominal aortic aneurysm, animal model, retroperitoneal approach, elastase, calcium chloride, beta aminopropionitrile

## Abstract

**Background:**

Constructing an ideal model of abdominal aortic aneurysm (AAA) is of great significance to elucidate its complex pathogenesis. Therefore, we introduce a new and simple method to simulate human AAA and construct a rat AAA model through a retroperitoneal approach.

**Methods:**

Forty healthy adult Sprague Dawley (SD) rats were randomly divided into a control group, elastase + calcium chloride group (PPE+CaCl_2_), elastase group (PPE), and elastase + beta aminopropionitrile group (PPE+BAPN) according to a male-female ratio of 1:1, with 10 rats in each group. A retroperitoneal approach was used to free the infrarenal abdominal aorta in all four groups. In the PPE + CaCl_2_ group, 0.1 ml of elastase (approximately 5 U) was perfused into the arterial cavity for 20 min, and 1.0 mol/L calcium chloride was infiltrated out of the arterial cavity for 10 min. In the PPE group, 0.1 mL of elastase (approximately 5U) was perfused into the arterial cavity for 20 min, and normal saline was infiltrated out of arterial cavity for 10 min; the PPE + BAPN group combined with 0.3% BAPN drinking water/day on the basis of PPE group; the control group was treated with saline instead of elastase and calcium chloride. Abdominal aortic specimens were collected after 4 weeks of feeding. The diagnostic criteria of AAA were 50% dilation of the abdominal aorta or rupture of the aneurysm at 4 weeks after the operation. Histopathology, immunohistochemistry, quantitative PCR (qPCR), western blotting assay, gelatine zymogram, and other methods were used.

**Results:**

The operation time of the four groups was controlled at approximately 40 min, and the success rate of the operation was 100%. Survival rate: Control Group (100%) = PPE Group (100%) > PPE + CaCl_2_ Group (90%) > PPE + BAPN Group (40%); Aneurysm formation rate: PPE + BAPN Group (100%) > PPE + CaCl_2_ Group (80%) > PPE Group (60%) > Control Group (0%); Aneurysm rupture rate: PPE + BAPN group (60%) > PPE + CaCl_2_ group (12.5%) > PPE group (0%);Inflammatory cells (macrophages, T cells, B cells, dendritic cells) infiltrated in different degrees in the PPE + CaCl_2_, PPE and PPE + BAPN groups. Vascular thickness, elastic fiber content, collagen fiber content, and vascular smooth muscle cell content in the PPE + CaCl_2_ group and PPE + BNPA group were significantly lower than those in Control group (*P* < 0.05). The content of elastic fibers and vascular smooth muscle cells in the PPE group were significantly lower than that in Control group (*P* < 0.05). The expression and activity of matrix metalloproteinase 2 (MMP2) and MMP9 in the PPE + CaCl_2_ group, PPE group, and PPE + BNPA group were significantly higher than those in the control group (*P* < 0.05).

**Conclusions:**

A new, simple, and reproducible rat AAA model can be constructed by a retroperitoneal approach. The pathological features of the three models are effective simulation of human AAA inflammatory cell infiltration, protease activity enhancement, and extracellular matrix destruction. The PPE+ CaCl_2_ model has the advantages of a high survival rate, high aneurysm formation rate, good stability, and reproducibility. It is an ideal animal model for studying the pathogenesis of AAA. The PPE + BAPN model can simulate the characteristics of spontaneous rupture of aneurysms. It is an ideal animal model to study the mechanism of AAA rupture.

## Background

Abdominal aortic aneurysm (AAA) is one of the most challenging cardiovascular diseases and usually has no symptoms but has the risk of spontaneous dilation and rupture. Once the aneurysm ruptures, the mortality rate is as high as 80% (1). Because of its potential lethality, AAA has always been a research hotspot. At present, the occurrence and development mechanism of AAA remains unclear. Therefore, it is of great significance to construct an ideal aneurysm model for studying the pathogenesis of AAA. A large number of studies have confirmed that the mixed chemical model based on high-pressure intra-arterial perfusion of elastase can better simulate the characteristics of inflammatory cell infiltration, endogenous protease activation, extracellular matrix degradation, smooth muscle cell apoptosis, and thrombosis, which is an ideal modeling method (2, 3). Unfortunately, this kind of modeling method is performed through an intraperitoneal approach, which destroys the integrity of the peritoneal cavity and loses extra body fluids and heat, which increases the chance of postoperative low-temperature reaction and abdominal infection (4). It is necessary to separate the peritoneum and retroperitoneum during the operation to prolong the operation time and increase the difficulty of the operation (5). The intestinal tract needs to be pulled during the operation, which is easily complicated by intestinal obstruction and increases postoperative complications (2). There are high mortality rates during the operation due to long-term and high-pressure perfusion and high disability rates due to lower limb ischemia after the operation (6). The difficulty of modeling, low success rate of operation, and high mortality during and after operation limit the wide application of this kind of model. However, the retroperitoneal approach can directly reach the abdominal aorta, avoiding the risk caused by the transabdominal approach. The retroperitoneal approach can be performed in the lateral position through a lumbar incision or prone position through a back incision. Therefore, in this study, a retroperitoneal approach was used to simplify the operation. At the same time, the models of elastase combined with calcium chloride, single elastase, and elastase combined with beta aminopropionitrile were compared, and it was expected to build a new rat AAA model with a high success rate, high aneurysm formation rate, low mortality rate, strong anthropomorphism, and replicability.

## Methods

### Rats and Ethics Statement

Forty healthy adult SD rats were purchased from the Laboratory Animal Center of North Sichuan Medical College. The experiments involving animals were approved by the Animal Care and Use Ethical Committee of General North Sichuan Medical College and complied with the Guide for the Care and Use of Laboratory Animals approved by the National Institutes of Health.

### Experimental Grouping

Forty rats were randomly divided into the control group, elastase + calcium chloride group (PPE + CaCl_2_), elastase group (PPE), and elastase + beta aminopropionitrile group (PPE + BAPN) according to a male-female ratio of 1:1, with 10 rats in each group. All the rats were fed for 4 weeks after the operation, and abdominal aorta specimens were collected by laparotomy 4 weeks after the operation.

### Establishment of Animal Model

#### PPE Model

① The rats were fasted 12 h before the operation and anesthetized with pentobarbital sodium at 40–50 mg/kg. The right lateral position was taken after anesthesia. Conventional skin preparation, disinfection, and towel laying occurred. The line from the costal edge of the left posterior axillary line to the thigh root was taken as an incision, with a length of approximately 3–3.5 cm. After cutting the skin, subcutaneous tissue, internal oblique muscles, external oblique muscles, and transverse abdominal fascia layer by layer, retroperitoneal adipose tissue was observed. The potential gap between the peritoneum and lumbar dorsal muscles could be found along the adipose tissue. Separating this gap with cotton swabs allowed us to reach the abdominal aorta directly. The abdominal aorta and inferior vena cava were separated carefully, and the 1 cm main abdominal aorta was dissociated below the left kidney. The diameter of the abdominal aorta under the kidney was measured with a Vernier caliper and recorded. The average value was measured by two experimenters.

**Figure 1:**
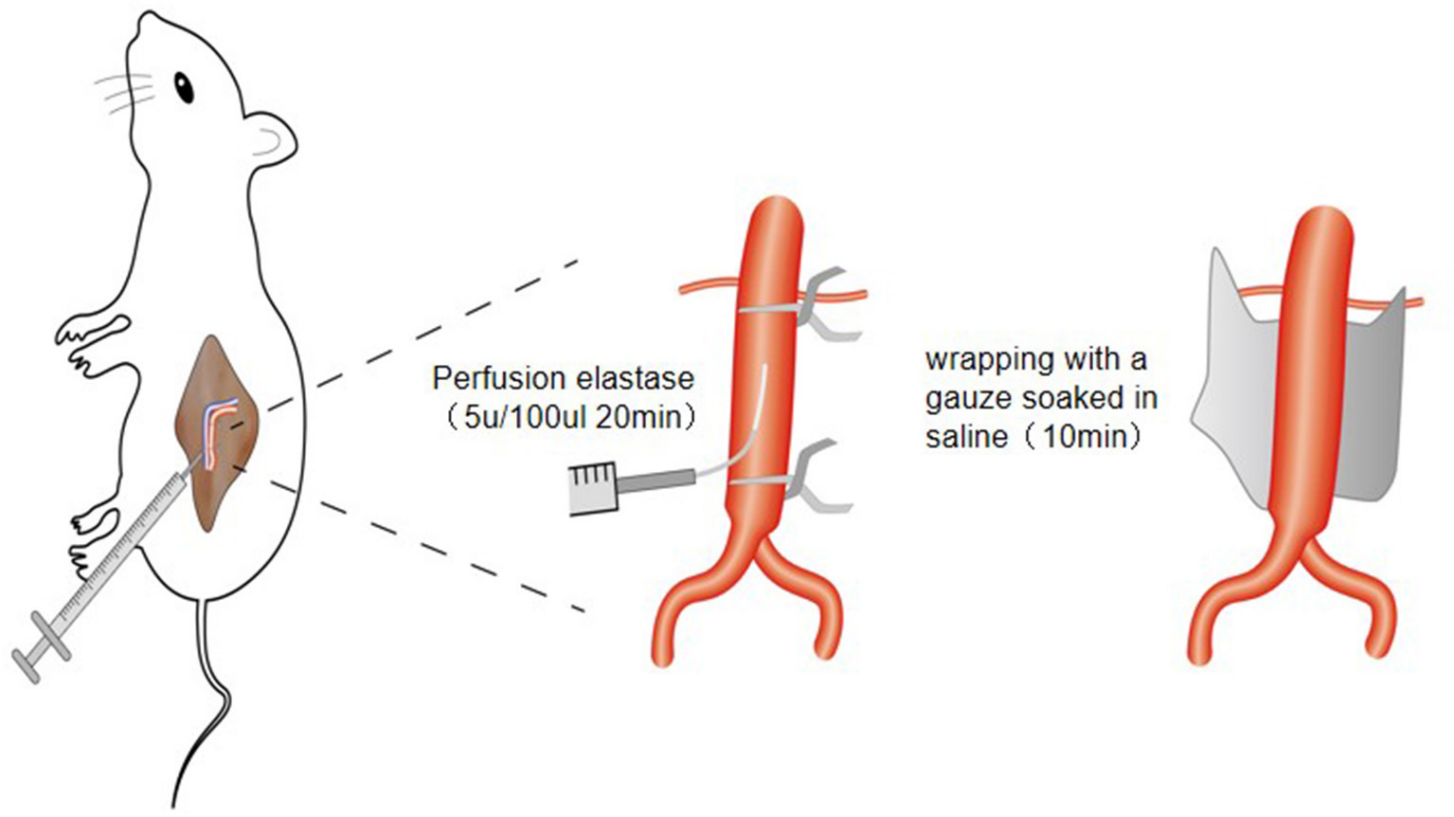
Schematic diagram of establishing animal model of abdominal aortic aneurysm in rats through retroperitoneal approach.

② The abdominal aortic branch of the perfusion segment was fully freed and ligated with mousse thread. The surgical field of vision was fully exposed with a mastoid distractor. First, the proximal end of the abdominal aorta was blocked with a microvascular clamp, then the blood in the perfusion section was squeezed to the distal end with a microvascular clamp, and the distal end of the abdominal aorta was blocked with a microvascular clamp (the distance between the two vascular clamps should be greater than 0.5 cm). The blood vessels collapsed completely, indicating that the blood vessels were well sealed. Then, 0.2 mL of elastase (approximately 10 U) was extracted with a disposable insulin syringe (1 mL 30G). The syringe needle was bent slightly with vascular forceps to make it slightly “L” shaped, and then used to puncture the abdominal aorta. After successful puncture, 0.1 mL of elastase (approximately 5 U) was slowly injected to fully fill the abdominal aorta in the perfusion segment. The puncture needle was fixed and maintained for 20 min. During this period, if there was little extravasation of elastase, it was supplemented in time to keep the abdominal aorta in a full filling state at all times. After the operation, the medicine was drawn in the blood tube, the needle was pulled out, the puncture point was covered with a gelatine sponge, and the tube was pressurized with a cotton ball. The distal vascular clip was removed first, followed by the proximal vascular clip. After observation for several minutes, no active bleeding was found. A small gauze soaked in normal saline was applied to the surface of the abdominal aorta in the perfusion section, and the small gauze was removed after 10 min. The retroperitoneal space was cleaned, checked for bleeding, and the incision closed layer by layer. After the operation, the rats were raised in a single cage and fed routinely (Figure 1).

#### PPE + CaCl_2_ Model

On the basis of the PPE model, the normal saline small gauze was replaced with a 1.0 mol/L sterile calcium chloride small gauze.

#### PPE + BAPN Model

Combined with 0.3% BAPN drinking water/day on the basis of the PPE model, the success rate, survival rate, aneurysm formation rate, and aneurysm rupture rate were calculated. Success rate of operation = number of rats surviving intraoperatively/total number of rats × 100%; Survival rate = number of surviving rats during and after operation/total number of rats × 100%; Aneurysm formation rate = number of rats meeting aneurysm standard/total number of rats surviving the operation × 100%; Aneurysm rupture rate = number of aneurysm ruptured rats/total number rats with aneurysms × 100%; Abdominal aortic dilatation rate = (abdominal aortic diameter 4 weeks after operation-abdominal aortic diameter before perfusion)/abdominal aortic diameter before perfusion × 100%, and abdominal aortic dilatation rate over 50% or aneurysm rupture 4 weeks after operation was taken as the standard for diagnosis of AAA (7).

### Hematoxylin and Eosin, Masson, Verhoeff's Van Gieson (EVG) Staining, and Immunohistochemical Detection

Paraffin-embedded 5-μm-thick sections were stained with hematoxylin and eosin (HE) for general histology. Masson staining and Verhoeff's Van Gieson (EVG) staining were used to evaluate the integrity of collagen fibers and elastic fibers of the abdominal aorta, respectively. To detect the target protein expression, primary antibodies against CD3 (1:1000; Proteintech), CD20 (1:100; Invitrogen), CD68 (1:1000; Proteintech), OX-62 (1:500; BD), and smooth muscle actin (SMA) (1:3000; Proteintech) were used. Each section was randomly selected from five visual fields under high power to count cells or measure the area of positive areas, with PBS as the negative control.

### Fluorescence Quantitative PCR (QPCR) Assay

The total RNA of the perfused abdominal aorta in each group was extracted according to the instructions of the TRIzol kit (Takara), and cDNA was synthesized by reverse transcription according to the instructions of the reverse transcription kit (Takara). The primer sequences are shown in Table 1. PCR conditions: the first step: predenaturation at 95°C 30 s for one cycle, PCR at 95°C 5 s → 60°C 30 s for 40 cycles, the second step: dissolution curve at 95°C 6 s → 65°C 5 s → 95°C 30 s for one cycle. Using β-Actin and GAPDH as reference genes, the relative expression level of target genes was calculated by extended ΔCt-method.

**Table 1:** Primer sequences for quantitative PCR (qPCR).

**Gene name**	**Forward primer (5′-3′)**	**Reverse primer (5′-3′)**
β-Actin	CAGGCATTGCTGACAGGATG	TGCTGATCCACATCTGCTGG
GAPDH	GCACCGTCAAGGCTGAGAAC	TGGTGAAGACGCCAGTGGA
MMP2	GACCCTGGAGCTTTGATGGC	GTGTAGGCGTGGGTCCAGTA
MMP9	GCTCGGATGGTTATCGCTGG	CCAGTTACAGTGACGTCGGC

### Western Blotting Assay

The aortic tissues were homogenized, and total protein was extracted. To detect the target protein expression, primary antibodies against matrix metalloproteinase 2 (MMP2) (1:1000 Proteintech), MMP9 (1:500 Proteintech), and GAPDH (1:20000 Proteintech) were used. After the images were collected by a Bio–Rad imaging system, the density of the gray value was analyzed by ImageJ, and the relative protein expression levels of MMP2 and MMP9 in the perfused abdominal aorta were calculated with GAPDH as the internal reference.

### Gelatine Zymogram

The activities of MMP-2 and MMP-9 in rat abdominal aortae were detected by a MMP Zymography Assay Kit (Applygen). The kit instructions were followed.

### Statistical Analysis

Statistical Product and Service Solutions (SPSS) 21 statistical analysis software was used to analyze the data. The measurement data were expressed by M (P25, P75). The Mann–Whitney test was used to compare the measurement data between the two groups, and the Kruskal–Wallis H test was used to compare the measurement data between multiple groups. The x^2^ test or Fisher's exact probability method was used for counting data. If *P* was < 0.05, the difference was statistically significant. ImageJ and Image-Pro Plus 6.0 software were used for image analysis and blood vessel parameter measurement.

## Results

### Success Rate of Operation and Survival Rate of Animals

The operation time of the four groups was controlled at approximately 40 min, and the success rate of the operation was 100%. There were no deaths during or after the operation in the control group and PPE group, and the survival rate was 100%. In the PPE + CaCl_2_ group, there were no deaths during the operation, and one rat died on the 7th day after the operation. The cause of death was aneurysm rupture and hemorrhage, and the survival rate was 90%. In the PPE + BAPN group, there were no deaths during the operation; one rat died on the 3rd, 10th, 21^st^, and 22nd days after the operation, and two rats died on the 26th day after the operation. The cause of death was rupture and hemorrhage of the aneurysm, and the survival rate was 40%.

### Dilatation Degree of the Abdominal Aorta, Aneurysm Formation Rate, and Aneurysm Rupture Rate

There was no significant difference in the diameter of the abdominal aorta before perfusion among the control group, PPE + CaCl_2_ group, PPE group, and PPE + BAPN group (*p* > 0.05). At 4 weeks after the operation, the diameter and dilatation rate of the perfusion abdominal aorta in the PPE group, PEE + CaCl_2_ group, and PPE + BNPA group were significantly larger than those in the control group (*P* < 0.05), but there was no significant difference among the other groups (*P* > 0.05). No aneurysm was found in the control group, and the aneurysm formation rate was 0%. In the PPE group, there were 6 aneurysms (4 males and 2 females, aneurysm formation rate = 60%), and there was no aneurysm rupture, the aneurysm rupture rate was 0%. In the PPE + CaCl2 group, 8 aneurysms were formed (5 males and 3 females, aneurysm formation rate = 80%), and one of the aneurysms ruptured; the rupture rate of aneurysms was 12.5%. In the PPE + BAPN group, 10 aneurysms were formed (5 males and 5 females, aneurysm formation rate = 100%), and six of the aneurysms ruptured, the rupture rate of aneurysms was 60% (Table 2, Figure 2).

**Table 2:** Diameter of abdominal aorta of rats in each group.

**Group**	**Number of rats**	**Preoperative diameter (mm)**	**Diameter when taking specimens (mm)**	**Diameter expansion rate (%)**	**Aneurysm formation rate (%)**	**Aneurysm rupture rate (%)**
Control group	10	1.20 (1.20, 1.30)	1.30 (1.28, 1.32)	8.01 (0.00, 8.33)	0 (0/10)	0 (0/0)
PPE+CaCL_2_ group	10	1.30 (1.15, 1.35)	2.20 (1.85, 3.90)^##^	116.67 (51.75, 200.00)^##^	80 (8/10)^##^	12.5 (1/8)
PPE group	10	1.25 (1.10, 1.40)	2.10 (1.68,3.25)^##^	68.94 (37.94, 132.14)^##^	60 (6/10)^#^	0 (0/6)
PPE+BNPA group	10	1.25 (1.13,1.45)	4.20 (3.40, 5.00)^##^	198.97 (177.40, 326.06)^##^	100 (10/10)^##^	60 (6/10)

*The measurement data were expressed by M (P25, P75); M, Median; P25, 1st quartile; P75, 3rd quartile*;

# *: P < 0.05*,

## *: P < 0.01 compared with the control group*.

**Figure 2:**
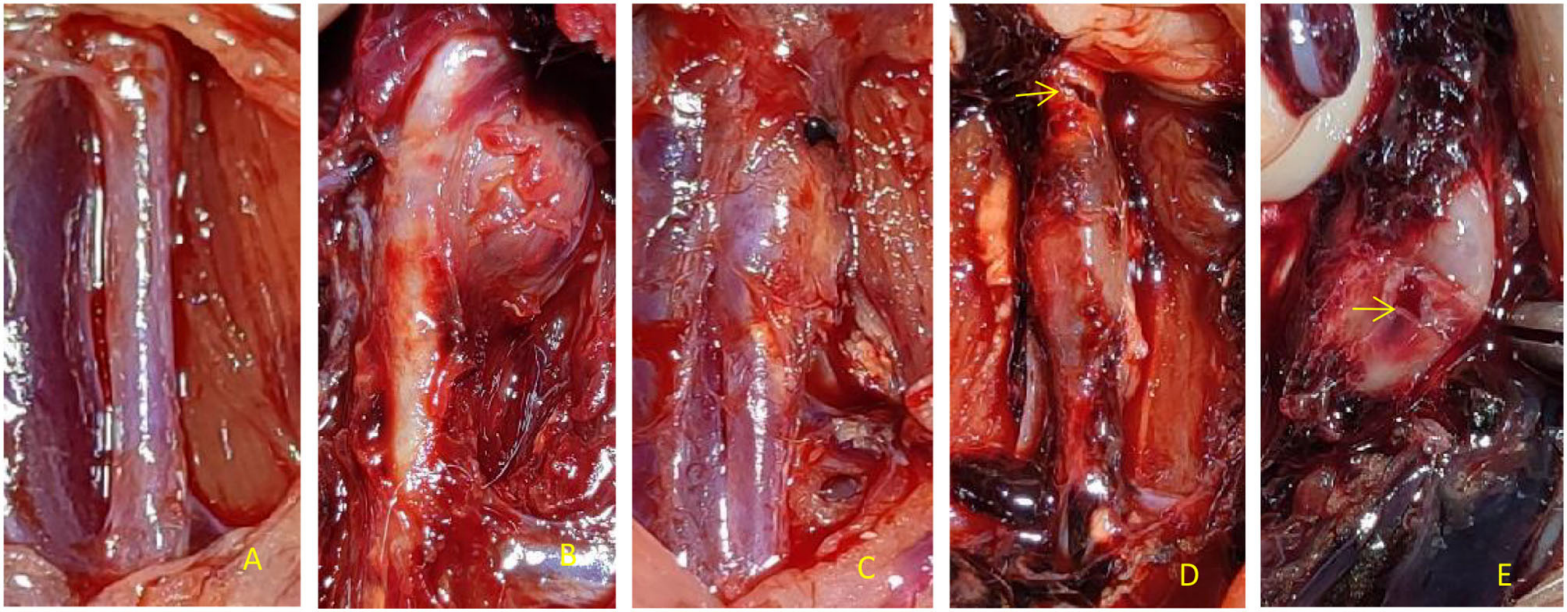
Dilatation degree and aneurysm appearance of the abdominal aorta in rats. **(A)** There was no dilation of abdominal aorta in perfusion segment in Control group, **(B)** PPE + CaCl_2_ group perfusion segment abdominal aortic saccular aneurysm local, **(C)** The fusiform aneurysm of abdominal aorta was perfused in PEE group, **(D)** In the PPE + BNPA group, the rupture of early aneurysm was in the anterior wall; **(E)** In the PPE + BNPA group, the rupture of late aneurysm was in the posterior wall.

### Morphological and Histological Changes in the Abdominal Aorta in the Perfusion Segment

The abdominal aortic aneurysms in the PPE group and PPE + BAPN group were mostly fusiform. All AAAs in the PPE + CaCl_2_ group were cystic. There were obvious adhesions around the abdominal aorta in the perfusion segment, and local calcifications could be seen. Microscopically, the vascular wall became thinner, the extracellular matrix of the media was degraded, some dissections and thrombosis were formed, the elastic fibers lost continuity, some were broken and missing, the smooth muscle cells decreased, and the inflammatory cells of the media and adventitia infiltrated. The control group did not have the above phenomenon. Compared with control group, the content of elastic fibers and smooth muscle cells in PPE + CaCl_2_, PPE, and PPE + BAPN groups decreased significantly (*P* < 0.05), the thickness of blood vessels and the content of collagen fibers in PPE + CaCl_2_ and PPE + BAPN groups decreased significantly (*P* < 0.05), but there was no significant difference in the thickness of blood vessels and the content of collagen fibers in PPE group (*P* > 0.05) (Table 3, Figure 3).

**Table 3:** Comparison of vascular thickness, elastic fiber content, collagen fiber content, and smooth muscle cell content in the abdominal aorta of rats in each group.

**Group**	**Number of rats**	**Thickness of blood vessel (μm)**	**Elastic fiber content** **(%)**	**Collagen fiber content (%)**	**Smooth muscle cell content (%)**
Control group	10	48.49 (40.27, 76.24)	32.21 (29.80, 41.80)	24.79 (22.33, 28.14)	35.37 (31.08, 37.94)
PPE+CaCL_2_ group	10	17.39 (13.66, 23.73)^*##*^	15.44 (10.27, 19.15)^*##*^	18.92 (16.22, 22.80)^#^	21.48 (17.28, 23.95)^*##*^
PPE group	10	28.42 (22.21, 39.56)	21.57 (11.68, 26.02)^*##*^	25.08 (22.64, 27.29)	23.76 (19.60, 25.59)^#^
PPE+BNPA group	10	14.78 (13.07, 15.66)^*##*^	10.75 (9.14, 13.57)^*##*^	16.64 (11.89,20.58)^#^	17.28 (15.66, 20.30)^*##*^

*The measurement data were expressed by M (P25, P75); M, Median; P25, 1st quartile; P75, 3rd quartile*;

# *: P < 0.05*,

## *: P < 0.01 compared with the control group*.

**Figure 3:**
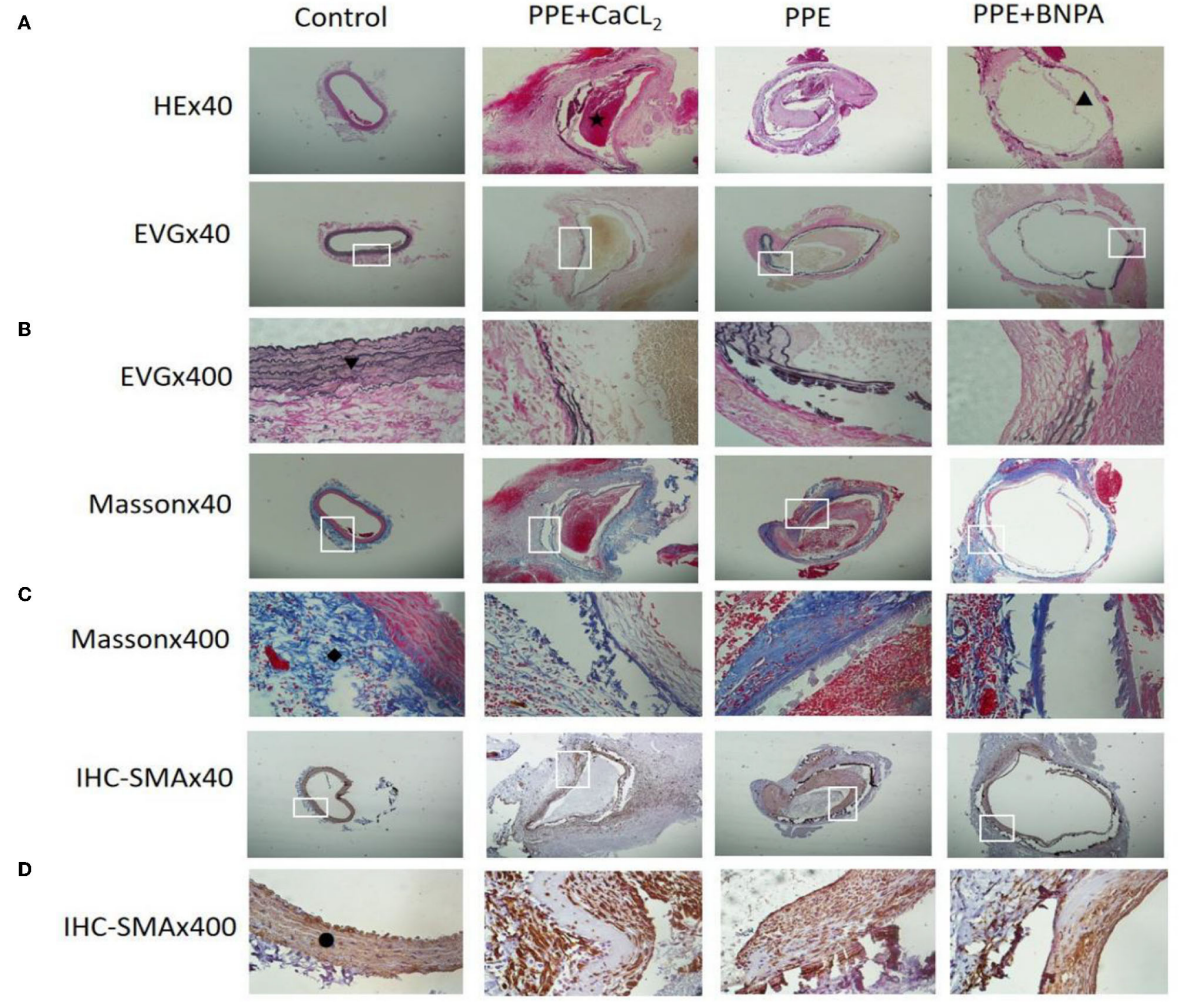
Histopathological changes of abdominal aortas in rats. **(A)** Representative pictures of HE staining in each group; **(B)** Representative pictures of EVG staining in each group; **(C)** Representative pictures of Masson staining in each group; **(D)** Representative pictures of SMA-IHC staining in each group. ⋆: Indicates thrombosis; ▴: Indicates interlayer; ▾: Indicates elastic fibre; ♦: Indicates collagen fibre; •: Indicates smooth muscle cells.

### Immunohistochemical Analysis of Inflammatory Cells in the Abdominal Aortic Wall

**Figure 4:**
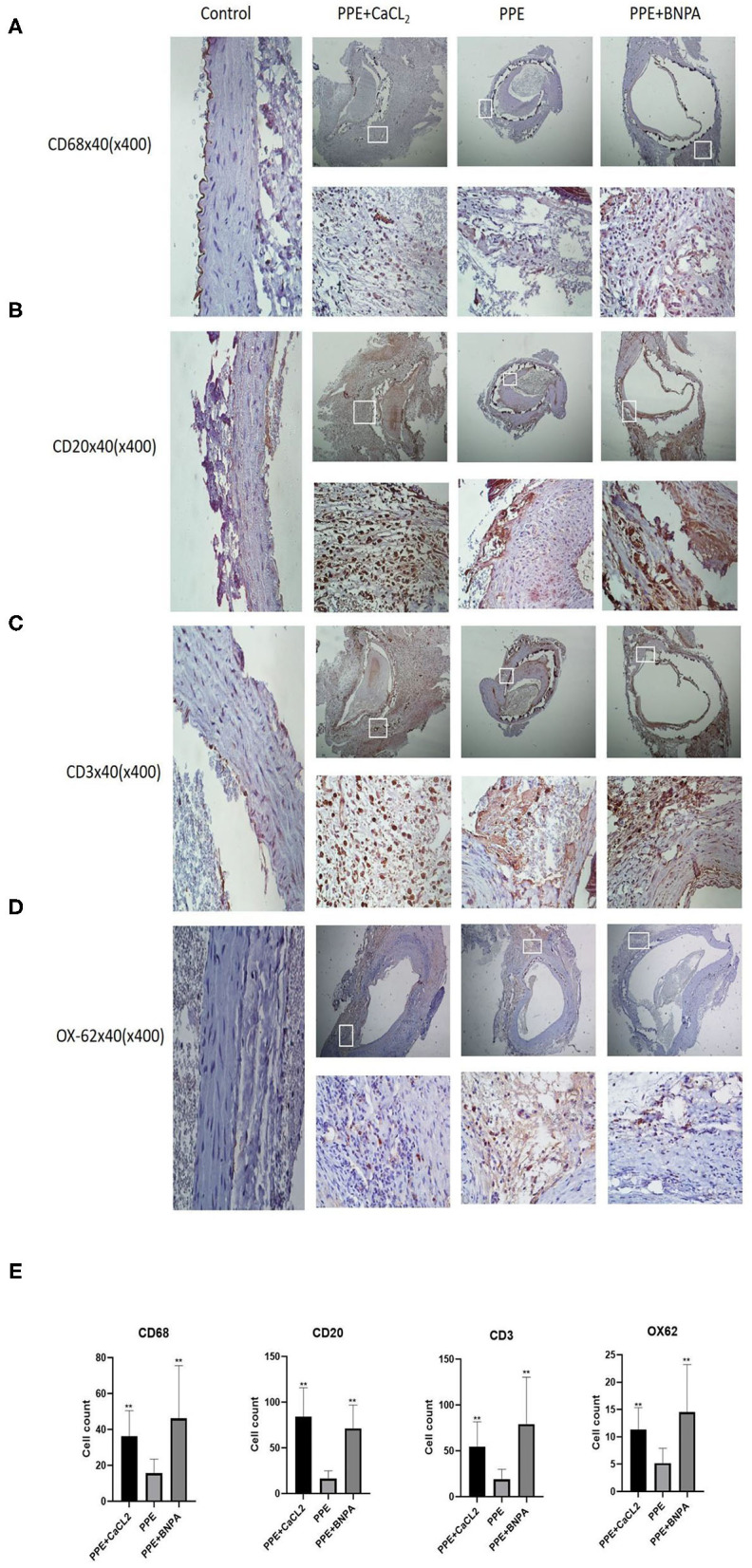
Infiltration of inflammatory cells in the abdominal aorta wall of rats. **(A–D)** Representative pictures of immunohistochemical (IHC) staining of CD68, CD20, CD3, and OX-62 cells; **(E)** CD68, CD20, CD3, and OX-62 cell count analysis. ^*^: *P* < 0.05, ^**^: *P* < 0.01 compared with the PPE group; *n* = 10.

There was no significant infiltration of inflammatory cells in the control group, but T cells, B cells, macrophages, and dendritic cells infiltrated to different degrees in the other three groups. The number of inflammatory cells in the PPE + CaCl_2_ group was as follows: B cells > T cells > macrophages > dendritic cells. The number of inflammatory cells in the PPE group and PPE + BAPN group was as follows: T cells > B cells > macrophages > dendritic cells. The number of T cells, B cells, macrophages, and dendritic cells in the PPE + BNPA group and PPE + CaCl_2_ group was significantly higher than those in PPE group (*P* < 0.05), but there was no significant difference in other groups (*P* > 0.05) (Figure 4).

### Expression and Activity of MMP2 and MMP9 in the Abdominal Aorta Wall

**Figure 5:**
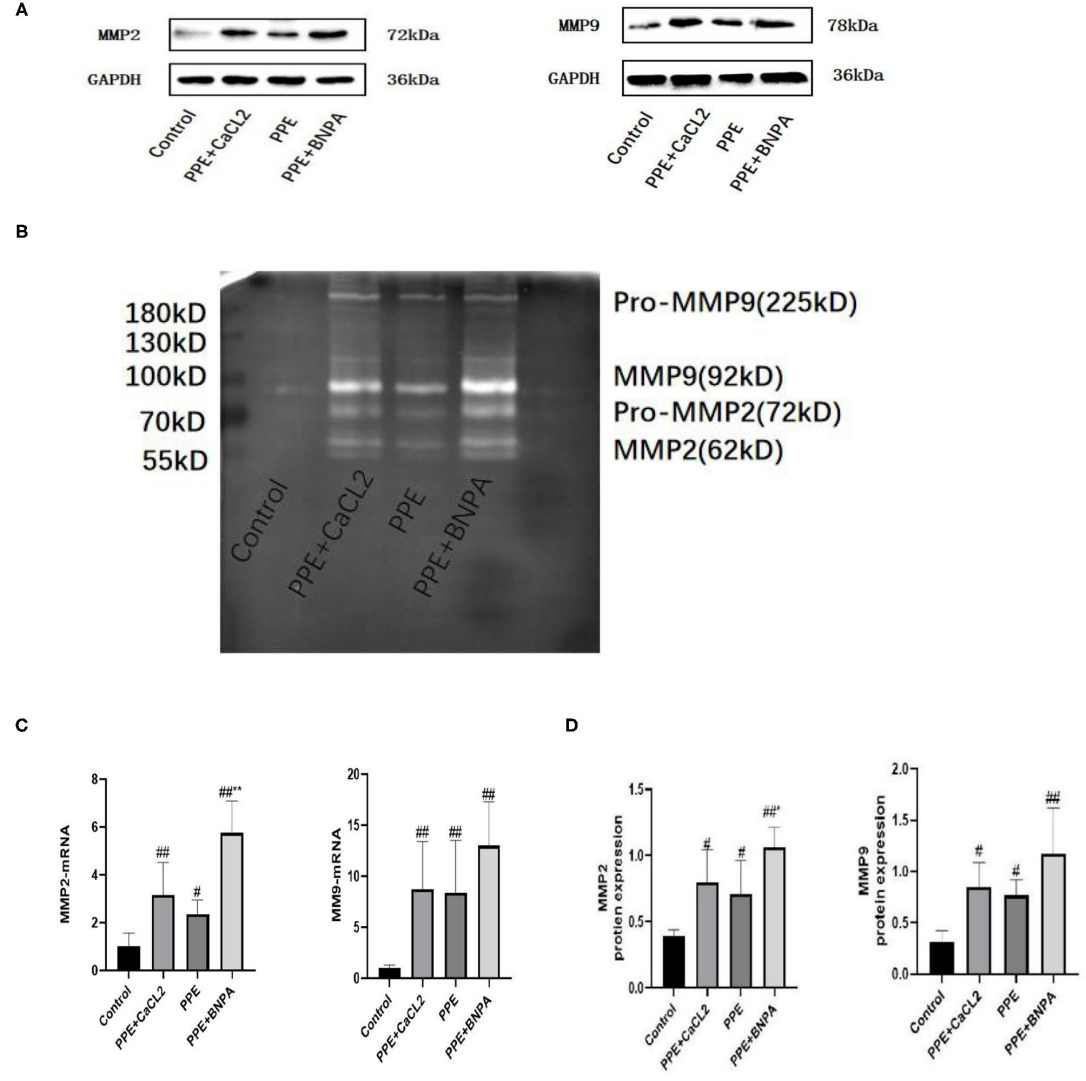
Expression and activity of MMP2 and MMP9 in the abdominal aorta wall of rats. **(A)** Typical Western blot analysis of MMP9 and MMP2 protein expression in each group; **(B)** Typical zymogram analysis of MMP9 and MMP2 protein activities in each group; **(C)** Quantitative analysis of relative expression of MMP2 and MMP9mRNA, *n* = 6; **(D)** Quantitative analysis of relative expression of MMP2 and MMP9 protein, *n* = 4; ^*^: *P* < 0.05, ^**^: *P* < 0.01 compared with the PPE group; #: *P* < 0.05, ##: *P* < 0.01 compared with the control group.

Compared with the control group, the expression of MMP2 and MMP9 mRNA and protein in the PPE + CaCl_2_ group, PPE group, and PPE + BAPN group increased significantly (*P* < 0.05), and the activity of MMP2 and MMP9 increased significantly (*P* < 0.05). Compared with the PPE group, the expression of MMP2 mRNA and protein in the PPE + BAPN group increased significantly (*P* < 0.05), and the activity of MMP2 increased significantly (*P* < 0.05). There was no significant difference in MMP2 mRNA, protein expression, or MMP2 activity among the other groups (*P* > 0.05). There was no significant difference in MMP9 mRNA or protein expression or MMP9 activity among the PPE + CaCl_2_ group, PPE group, and PPE + BAPN group (*P* > 0.05) (Figure 5).

## Discussions

At present, the pathogenesis of AAA is not clear. Constructing a simple and reliable animal model of AAA provides a theoretical and practical basis for studying the pathogenesis and treatment of AAA. The animal models of AAA can be divided into three categories: physical, chemical, and gene induction methods, among which the chemical induction (angiotensin II, elastase, calcium chloride, beta aminopropionitrile) models are mainly used to study the pathogenesis (8).

The pressurized perfusion of elastase established by Anidjar et al. (6) is one of the most commonly used chemical modeling methods at present and simulates the degradation process of extracellular matrix elastase during the formation of AAA. Although a large number of reports prove the feasibility of this method, this method still has many shortcomings. This method adopts an abdominal approach. The operation is complicated, the integrity of the peritoneal cavity is destroyed, and the body fluids and heat are lost, which increases the risk of postoperative hypothermia reaction and abdominal infection. The intestinal tract should be pulled during the operation, which easily causes intestinal obstruction and injury to abdominal organs. The peritoneum and retroperitoneum should be separated to prolong the operation time and increase the risk of death. For a long time (30 min-2 h), high pressure (>100 mmHg) and high dose (12 U) of elastase perfusion led to an intraoperative and postoperative mortality rate of 40% (6, 9–11). In this study, the surgical path and some details were improved on the basis of the classical elastase model. ① The retroperitoneal approach is a common surgical method in urology (12). Because the abdominal aorta and kidney are adjacent to each other and belong to extraperitoneal organs, it is possible to construct a rat model of infrarenal AAA through a retroperitoneal approach. The advantage of the retroperitoneal approach is that it can reach the abdominal aorta directly. There was no interference from internal organs, and the operation was relatively simple. It can avoid the risk caused by the intraperitoneal approach and significantly improve the success rate of the operation. However, there are the following limitations: (a) The retroperitoneal space is an unnatural space and lacks body surface markers. Therefore, it is more difficult to find the surgical entrance than traditional abdominal surgery. (b) The retroperitoneal space is narrow, which limits visual field exposure and operating space, and is difficult to completely free and expose the entire segment of the abdominal aorta. ② It is not necessary to completely free and expose the whole infrarenal abdominal aorta and ensure that the perfusion abdominal aorta is larger than 0.5 cm. Regardless of which approach is adopted, the excessive free and exposed blood vessels will increase the side injury and lead to massive hemorrhage, or local tissue and spinal cord ischemia due to ligation of too many branches and lumbar arteries, which will increase the risk of death in rats. ③ Classical elastase perfusion needs to maintain a perfusion pressure of 100 mmHg or higher by hydraulic or micropump syringe, but it is difficult to accurately reach and maintain the same pressure. Excessive pressure can cause elastase to enter the circulation and lead to the death of rats (10, 13, 14). After perfusion, it is necessary to ligate the punctured iliac artery or femoral artery, which is prone to unilateral limb ischemia and necrosis, and the disability rate is high (2, 6). Although there are reports that the puncture site is changed to the abdominal aorta, after perfusion, the absorbable vascular sutures are replaced to repair the puncture site of the abdominal aorta and restore the original anatomical structure of the abdominal aorta (15). This method requires microsurgical technology, which is difficult to operate, and the operator needs a certain vascular surgical foundation, which limits its wide application. The pressurized perfusion was not used in this study because there is no external mechanical pressure in the formation of human AAA (2). It is only necessary to slowly inject elastase into the abdominal aorta of the perfusion section to keep it full. The selected puncture needle was an insulin needle (30G), which causes minimal damage to blood vessels. After perfusion, local compression can stop bleeding. The success rate of operation is significantly improved, and the model is more in line with the formation process of human AAA. This method is simple and easy to use, without the help of microsurgical techniques and expensive instruments such as micropumps and is easy to popularize. ④ The dosage of elastase was reduced (5 U), and an excessive dosage of elastase easily led to the death of rats (14). ⑤ The perfusion time was shortened (20 min). The classical elastase perfusion time is 2 h. Too long of a time to block the abdominal aorta easily forms thrombi, and a long-term concentration of blood above the blocking site easily leads to brain oedema and lower limb ischemia, with high mortality (16). Sinha et al. (17) shortened the perfusion time to 30 min and successfully established AAA, which significantly reduced the occurrence of brain oedema and mortality, but there remained a certain proportion of lower limb ischemia incidence. In this study, the perfusion time was shortened to 20 min, and no lower limb ischemia occurred.

This study confirmed that it is feasible to construct a rat elastase model through a retroperitoneal approach. However, the aneurysm formation rate is only 60%, which is related to the perfusion of elastase at only 0.1 mL (5 U). To further improve the aneurysm formation rate, we should consider moderately increasing the perfusion amount and concentration of elastase and prolonging the perfusion time, but these changes may lead to an increase in mortality. In this study, to avoid affecting the survival rate while further increasing the rate of aneurysm formation, two methods of adding calcium chloride and beta aminopropionitrile to elastase perfusion were investigated separately. Calcium chloride (18) induced the inflammatory reaction of the arterial wall, while beta aminopropionitrile (19) inhibited the cross-linking process of collagen and elastin to construct an AAA animal model. Using these two methods alone has the disadvantage of a low aneurysm formation rate. Therefore, in this study, two chemical induction methods were combined to construct an AAA animal model to improve the rate of aneurysm formation. In this study, in the elastase combined with calcium chloride group, the success rate of the operation was 100%, the survival rate was 90%, the total aneurysm formation rate was 80%, the male aneurysm formation rate was 100%, and the female aneurysm formation rate was 60%. Consistent with the epidemiological investigation of human AAA, the incidence rate of males was higher than that of females (20). The model has good stability and is suitable for studying the pathogenesis of AAA. In the elastase combined with beta aminopropionitrile group, although the formation rate of aneurysms was 100%, the rupture rate of aneurysms was as high as 60%. The model has poor stability and is suitable for the study of dynamic AAA fracture mechanisms. There is also an angiotensin II (21) model that can simulate the unique characteristic atherosclerosis phenomenon of human beings, but it needs to use gene knockout mice, which has limited sources, strict requirements on animal feeding and reproduction conditions, and certain restrictions on experimental research. Therefore, this study did not use angiotensin II for discussion.

In addition, the present study compared the morphology and pathology of the three models. The aneurysms in the Elastase Group and Elastase Combined with beta aminopropionitrile Group were mainly spindle-shaped, which was consistent with most AAAs found in human pathology. The aneurysms in the elastase and calcium chloride groups were all saccular. The synergistic effect of elastase and calcium chloride stimulates the inflammatory reaction and elastolytic cascade reaction of the abdominal aorta wall. The aneurysms in the three models were characterized by disordered arterial wall structure, thinning of the media, serious degradation of elastic fibers, loss of continuity or even disappearance, reduction of nucleated smooth muscle cells in the media, and dissection with mural thrombosis in some of the aneurysms. Inflammatory cells (macrophages, dendritic cells, T cells, and B cells) infiltrated to different degrees in both media and adventitia, especially T and B cells. However, the classical elastase model is dominated by macrophage infiltration (22, 23). This difference occurs because the time nodes observed are different. Macrophages mediate the innate immune response, which occurs immediately in the early stage of disease. T and B cells mediate the adaptive immune response, which occurs relatively late but runs through the disease all the time. The time node observed in this study is the 4th week, which is the late model, while the time node observed in the classical elastase model is the 1st week and the 2nd week, which is the early model. The infiltration of inflammatory cells in the elastase combined with calcium chloride group and elastase combined with beta aminopropionitrile group was greater than that in the elastase group. Inflammatory cells are the main source of MMPs in the arterial wall, and MMP2 and MMP9 are important proteases that degrade the extracellular matrix of arterioles, which are the main causes of AAA (24). The results of this study also confirmed that the expression and activity of MMP2 and MMP9 were significantly increased in the three groups of aneurysms. It is worth mentioning that the expression and activity of MMP2 in the elastase combined with beta aminopropionitrile group (aneurysm rupture group) were significantly higher than those in the elastase group (no aneurysm rupture group), and the content of elastic and collagen fibers decreased most significantly in the three groups of models. The existing studies have shown that MMP2 degrades both elastic and collagen fibers, and the degradation of elastic fibers is associated with aneurysm dilation, while the degradation of collagen fibers is associated with aneurysm rupture (25). It can be concluded that MMP2 may play an important role in aneurysm rupture. This result is consistent with the research results of Lu et al. (26) and Sean et al. (27) believed that the increased activity of MMP9 was closely related to aneurysm rupture. The reasons for the different conclusions may be the different methods adopted by researchers, the different observation time nodes, and the great individual differences of the specimens taken.

## Conclusions

Through the change of surgical approach and the improvement of intraoperative details, the mortality of experimental animals was obviously reduced, and the survival rate was improved. In this study, the elastase combined with calcium chloride model and the elastase combined with beta aminopropionitrile model were similar to human AAA in histomorphology, inflammatory cell infiltration, and vascular matrix destruction. Elastase combined with calcium chloride has the advantages of a high survival rate, high tumorigenic rate, good stability, and reproducibility. It is an ideal animal model for studying the pathogenesis of AAA. Elastase combined with a beta aminopropionitrile model can simulate the characteristics of spontaneous rupture of aneurysms. It is an ideal animal model to study the mechanism of AAA rupture.

## Data Availability Statement

The original contributions presented in the study are included in the article/supplementary material, further inquiries can be directed to the corresponding author/s.

## Ethics Statement

The animal study was reviewed and approved by the Institutional Animal Care and Use Ethical Committee of North Sichuan Medical college. Written informed consent was obtained from the owners for the participation of their animals in this study.

## Author Contributions

J-XZ performed experiments, interpreted the data, and wrote the manuscript. YY conceived the research design, experimental plan, and manuscript revision. Q-QT participated in the experiment. X-CS, S-YY, and CZ participated in the data analysis. All the authors read and approved the final draft.

## Funding

This work was supported by the Sichuan Province Science and Technology Planning Project (2016JY0172) and the Scientific Research Project of Sichuan Provincial Health and Family Planning Commission (16PJ121).

## Conflict of Interest

The authors declare that the research was conducted in the absence of any commercial or financial relationships that could be construed as a potential conflict of interest.

## Publisher's Note

All claims expressed in this article are solely those of the authors and do not necessarily represent those of their affiliated organizations, or those of the publisher, the editors and the reviewers. Any product that may be evaluated in this article, or claim that may be made by its manufacturer, is not guaranteed or endorsed by the publisher.

## Abbreviations

AAA: abdominal aortic aneurysm
PPE+CaCL_2_: elastase + calcium chloride
PPE: elastase
PPE+BAPN: elastase + beta aminopropionitrile
MMPs: matrix metalloproteinases.

## References

[1] Lattanzi S. Abdominal aortic aneurysms: pathophysiology and clinical issues. J Intern Med. (2020) 288:376–8. 10.1111/joim.13060 32301175

[2] Tanaka A Hasegawa T Chen Z Okita Y Okada KA. novel rat model of abdominal aortic aneurysm using a combination of intraluminal elastase infusion and extraluminal calcium chloride exposure. J Vasc Surg. (2009) 50:1423–32. 10.1016/j.jvs.2009.08.062 19958989

[3] Bernal Uribe S Lopez-Sanz L Melgar A La-Manna S Jimenez-Castilla L Prieto I. P3114Protective effect of SOCS1-based therapy in experimental abdominal aortic aneurysm. Eur Heart J. (2019) 40:ehz745–0189. 10.1093/eurheartj/ehz745.0189

[4] Liu Z Wang Q Ren J Assa CR Morgan S Giles J. Murine abdominal aortic aneurysm model by orthotopic allograft transplantation of elastase-treated abdominal aorta. J Vasc Surg. (2015) 62:1607–14. 10.1016/j.jvs.2014.05.019 24974783 PMC4277509

[5] Busch A Chernogubova E Jin H Meurer F Eckstein HH Kim M. Four surgical modifications to the classic elastase perfusion aneurysm model enable haemodynamic alterations and extended elastase perfusion. J Vasc Surg. (2018) 68:102–9. 10.1016/j.ejvs.2018.03.018 29703523

[6] Anidjar S Salzmann JL Gentric D Lagneau P Camilleri JP Michel JB. Elastase-induced experimental aneurysms in rats. Circulation. (1990) 82. 10.1161/01.CIR.82.3.973 2144219

[7] Schack AS Stubbe J Steffensen LB Mahmoud H Laursen MS Lindholt JS. Intraluminal infusion of Penta-Galloyl Glucose reduces abdominal aortic aneurysm development in the elastase rat model. PLoS ONE. (2020) 15:e0234409. 10.1371/journal.pone.0234409 32857766 PMC7454949

[8] Murali Krishna S Morton SK Li J Golledge J. Risk factors and mouse models of abdominal aortic aneurysm rupture. Int J Mol Sci. (2020) 21:7250. 10.3390/ijms21197250 PMC7583758 33008131

[9] Carsten III CG Calton WC Johanning JM Armstrong PJ Franklin DP Carey DJ. Elastase is not sufficient to induce experimental abdominal aortic aneurysms. J Vasc Surg. (2001) 33:1255–62. 10.1067/mva.2001.112706 11389426

[10] Yue J Yin L Shen J Liu Z. A modified murine abdominal aortic aneurysm rupture model using elastase perfusion and angiotensin II infusion. Ann Vasc Surg. (2020) 67:474–81. 10.1016/j.avsg.2020.03.002 32171859

[11] Yamaguchi T Yokokawa M Suzuki M Higashide S Katoh Y Sugiyama S. Shortened elastase infusion time in the elastase-induced rat aneurysm model. J Surg Res. (1999) 85:158–62. 10.1006/jsre.1999.5622 10383853

[12] Prudhomme T Roumiguié M Gas J Soulié M Thoulouzan M Huyghe E. Comparison between retroperitoneal and transperitoneal laparoscopic adrenalectomy: are both equally safe? J Visc Surg. (2020) 158:204–10. 10.1016/j.jviscsurg.2020.07.009 32773296

[13] Azuma J Asagami T Dalman R Tsao PS. Creation of murine experimental abdominal aortic aneurysms with elastase. J Visual Exper. (2009) e1280. 10.3791/1280 PMC3148686 19629030

[14] Yamaguchi T Yokokawa M Suzuki M Higashide S Katoh Y Sugiyama S. Factors influencing mortality in the rat elastase-induced-aneurysm model. J Surg Res. (2000) 94:81–3. 10.1006/jsre.2000.5995 11104646

[15] Hu G Dong Z Fu W. A novel modification of the murine elastase infusion model of abdominal aortic aneurysm formation. Ann Vasc Surg. (2017) 42:246–53. 10.1016/j.avsg.2017.01.006 28288888

[16] Guarini S. A highly reproducible model of arterial thrombosis in rats. J Pharmacol Toxicol Method. (1996) 35:101–5. 10.1016/1056-8719(96)00006-8 8729436

[17] Sinha I Cho BS Roelofs KJ Stanley JC Henke PK Upchurch GR. Female gender attenuates cytokine and chemokine expression and leukocyte recruitment in experimental rodent abdominal aortic aneurysms. Ann N Y Acad Sci. (2006) 1085:367–79. 10.1196/annals.1383.027 17182958

[18] Xu T Wang S Li X Li X Qu K Tong H Zhang R. Lithium chloride represses abdominal aortic aneurysm via regulating GSK3β/SIRT1/NF-κB signaling pathway. Free Rad Biol Med. (2021) 166:1–10. 10.1016/j.freeradbiomed.2021.02.007 33588051

[19] Zheng HQ Rong JB Ye FM Xu YC Lu HS Wang JA. Induction of thoracic aortic dissection: a mini-review of β-aminopropionitrile-related mouse models. J Zhejiang Univ Sci B. (2020) 21:603–10. 10.1631/jzus.B2000022 32748576 PMC7445087

[20] Fashandi AZ Spinosa M Salmon M Su G Montgomery W Mast A Lu G. Female mice exhibit abdominal aortic aneurysm protection in an established rupture model. J Surg Res. (2020) 247:387–96. 10.1016/j.jss.2019.10.004 31699539 PMC7111562

[21] Adelsperger AR Phillips EH Ibriga HS Craig BA Green LA Murphy MP. Development and growth trends in angiotensin II-induced murine dissecting abdominal aortic aneurysms. Physiol Rep. (2018) 6:e13668. 10.14814/phy2.13668 29696811 PMC5917066

[22] Liu R Huang J Ge Y Liu S Huang T Cai H. Inhibition of phosphatidylinositol 3-kinase γ by IPI-549 attenuates abdominal aortic aneurysm formation in mice. Eur J Vasc Endovasc Surg. (2020) 60:254–63. 10.1016/j.ejvs.2020.03.042 32423743

[23] Xu B Iida Y Glover KJ Ge Y Wang Y Xuan H. Inhibition of VEGF (Vascular Endothelial Growth Factor)-A or its receptor activity suppresses experimental aneurysm progression in the aortic elastase infusion model. Arterioscler Thromb Vasc Biol. (2019) 39:1652–66. 10.1161/ATVBAHA.119.312497 31294623 PMC6699755

[24] Paghdar S Khan TM Patel NP Chandrasekaran S De Sousa JF Tsouklidis N. Doxycycline therapy for abdominal aortic aneurysm: inhibitory effect on matrix metalloproteinases. Cureus. (2021) 13. 10.7759/cureus.14966 PMC8191685 34123662

[25] Freestone T Turner RJ Coady A Higman DJ Greenhalgh RM Powell JT. Inflammation and matrix metalloproteinases in the enlarging abdominal aortic aneurysm. Arterioscler Thromb Vasc Biol. (1995) 15:1145–51. 10.1161/01.ATV.15.8.1145 7627708

[26] Lu G Su G Davis JP Schaheen B Downs E Roy RJ. A novel chronic advanced stage abdominal aortic aneurysm murine model. J Vasc Surg. (2016) 66:232–42. 10.1016/j.jvs.2016.07.105 PMC5483384 28274752

[27] English SJ Piert MR Diaz JA Gordon D Ghosh A D'Alecy LG. Increased 18F-FDG uptake is predictive of rupture in a novel rat abdominal aortic aneurysm rupture model. Ann Surg. (2015) 261:395. 10.1097/SLA.0000000000000602 24651130 PMC4662083

